# Acute ischemic stroke, active malignancy and non-bacterial thrombotic endocarditis: an exploratory prospective observational study

**DOI:** 10.1038/s41598-026-61415-8

**Published:** 2026-07-15

**Authors:** Athanasios Frydas, Ela Marie Akay, Hong Ran, Kudrat Rakhimov, Sarah Ouldali, Mazen Merhi, Frieder Pfäfflin, Linda Jürgens, Wolfram Doehner, Frank Tacke, Dominik Paul Modest, Anne Flörcken, Ingo Hilgendorf, Daniel Armando Morris, Eberhard Siebert, Christoph Leithner, Maximilian Schoels, Matthias Schneider-Reigbert

**Affiliations:** 1 Deutsches Herzzentrum der Charité, Department of Cardiology, Angiology and Intensive Care Medicine, Berlin, Germany; 2 Charité-Universitätsmedizin Berlin, Corporate Member of Freie Universität Berlin and Humboldt-Universität Zu Berlin, Berlin, Germany; 3 DZHK (German Center for Cardiovascular Research), Partner Site Berlin, Berlin, Germany; 4 Department of Cardiology, University Hospital Zurich, Zurich, Switzerland; 5 Department of Neurology, Charité, Berlin, Germany; 6 Nanjing First Hospital, Nanjing Medical University, Nanjing, China; 7 Department of Infectious Diseases and Respiratory Medicine, Charité, Berlin, Germany; 8 Berlin Institute of Health Center for Regenerative Therapies, Charité-Universitätsmedizin Berlin, Berlin, Germany; 9 Department of Gastroenterology, Charité, Berlin, Germany; 10 Charité Comprehensive Cancer Center, Charité, Berlin, Germany; 11 Department of Hematology, Oncology and Cancer Immunology, Charité, Berlin, Germany; 12 Institute for Neuroradiology, Charité, Berlin, Germany; 13 Berlin Institute of Health, Berlin, Germany; 14 Center for Stroke Research Berlin, Berlin, Germany; 15 Department of Internal Medicine and Cardiology, Deutsches Herzzentrum der Charité, Augustenburger Platz 1, 13353 Berlin, Germany

**Keywords:** Marantic endocarditis, NBTE, Echocardiography, Stroke, Thromboembolism, Cancer, Cardiology, Diseases, Medical research, Oncology

## Abstract

About 3–10% of patients with acute ischemic stroke (AIS) have active cancer. Malignancy-associated hypercoagulability (MAH) is an established cause of cancer-associated stroke. Nonbacterial thrombotic endocarditis (NBTE) is a severe manifestation of MAH. However, no prospective study has examined its prevalence and anticoagulation management in AIS. We conducted a prospective observational study at a tertiary center including patients with AIS or transient ischemic attack (TIA) and active malignancy. All patients underwent transthoracic echocardiography, followed by transesophageal echocardiography when indicated. Among 3,491 screened patients, 16 with active cancer and AIS/TIA were included. Eleven (68.8%) showed embolic patterns suggestive of MAH, and 6 (37.5%) had NBTE. NBTE patients numerically more frequently had multiterritory embolic infarctions (100% vs. 60.0%, p = 0.102) and prior ischemic stroke (66.7% vs. 10.0%, p = 0.028). 4/6 were on direct oral anticoagulants (DOACs), while none were on low-molecularweight heparin (LMWH). Under LMWH, vegetations resolved or regressed in 3/4, whereas under DOACs, progression or recurrent embolism occurred in several patients. Twelve-month mortality was high in both MAH (90.9%) and NBTE (83.3%) groups. NBTE was frequently identified in this prospective cohort of patients with cancer-associated stroke and consistently associated with multi-territory embolic infarction. Recognition may enable earlier diagnosis. Larger studies are needed to define optimal anticoagulation strategies.

## Introduction

Approximately 3–10% of patients presenting with acute ischemic stroke (AIS) have an active malignancy; this proportion increases to 10–15% when prior malignancy is also included. In patients with cryptogenic stroke, the proportion is even higher, reaching up to 20%^1,2^. Prevalence of cancer-associated stroke is likely to rise as cancer-specific survival improves with modern therapies. While cancer can be a bystander, especially in older adults, prospective data link incident malignancy to AIS^3^. Characteristic imaging patterns, such as multi-territorial or metachronous infarcts, often suggest a proximal embolic mechanism, with malignancy-associated hypercoagulability (MAH) being a likely explanation^4^. However, in the absence of additional arterial or venous thrombotic events (VTE) and without established biomarkers, MAH can be difficult to pinpoint, leaving clinicians in doubt whether to start anticoagulation.

Nonbacterial thrombotic endocarditis (NBTE), also known as “marantic endocarditis”, is considered a severe manifestation of MAH that is detectable on echocardiography and represents a potential embolic source. It is characterized by sterile platelet–fibrin vegetations that typically affect the mitral and aortic valves^5^. Its pathogenesis reflects tumor-induced coagulopathy and systemic inflammation, with overexpression of tissue factor, cytokines, and circulating procoagulants that promote platelet activation and thrombin generation. Endothelial injury and turbulent flow facilitate deposition of platelet–fibrin aggregates, which may embolize to the cerebral or systemic circulation^6,7^.

Although NBTE can occur in autoimmune disease (e.g., systemic lupus erythematosus, antiphospholipid syndrome), it most commonly affects patients with advanced malignancy—particularly adenocarcinomas of the lung, pancreas, and gastrointestinal tract^6,7^. While generally considered uncommon even in cancer patients^8^, retrospective data suggests a prevalence of 3–25% in patients with AIS and active malignancy^9–12^. Diagnosis is frequently delayed or missed, as NBTE lacks systemic infection markers and echocardiographic findings may be subtle. Often, transesophageal rather than transthoracic echocardiography is required to identify characteristic sterile vegetations. Moreover, the lack of consensus diagnostic criteria further complicates clinical recognition^6–8^.

In the absence of randomized controlled trials, optimal anticoagulation remains uncertain. Heparin therapy is traditionally recommended, and several reports describe recurrent embolism or failure of vegetation regression under direct oral anticoagulants (DOACs) in NBTE, suggesting medication-class-dependent differences in efficacy^6,7,13,14^.

As cancer-specific survival improves with modern oncologic therapies, recognition of cerebrovascular complications such as MAH and NBTE is becoming increasingly relevant in routine clinical practice. Despite its potential therapeutic relevance, prospective data on NBTE’s prevalence, presentation, and optimal management in cancer-associated stroke are lacking. We therefore conducted a single-center, prospective observational study to address this gap (Graphical abstract).

## Methods

### Ethicals

The study was conducted in accordance with the Declaration of Helsinki and approved by the Institutional Ethics Committee of Charité – Universitätsmedizin Berlin (approval number EA1/102/23). Written informed consent was obtained from all patients.

### Study design and patient population

Between December 2023 and December 2024, we prospectively enrolled consecutive adult patients admitted to our tertiary care university medical center with AIS or transient ischemic attack (TIA) who had active malignancy. Active malignancy was defined as:

1. metastatic or locally advanced cancer, or
2. receipt of systemic anticancer therapy (chemotherapy, immunotherapy, targeted or endocrine therapy), radiotherapy, or cancer surgery within the preceding 6 months, or
3. a newly diagnosed malignancy identified during hospitalization as the cause of the index cerebrovascular event.

Patients considered cured after completion of prior cancer-specific therapy, with no evidence of active disease, were excluded.

Follow-up was performed over a 12-month period.

### Neuroimaging assessment

All patients underwent magnetic resonance imaging (MRI) of the brain. In cases of acute-onset focal neurologic deficit, computed tomography angiography (CTA) of the intracranial and extracranial arteries was performed immediately on presentation, with MRI obtained subsequently as soon as clinically feasible. Brain MRI was reviewed, and infarct patterns were categorized by vascular territory involvement (one, two, or three territories), circulation (anterior vs. posterior), laterality (unilateral vs. bilateral), lesion count (none vs. single vs. multiple), and timing (metachronous vs. synchronous).

### Additional stroke work-up

Additional stroke work-up was conducted per local standards at the treating physician’s discretion, typically including sonography of the extracranial vessels, cardiac rhythm monitoring for atrial fibrillation, and laboratory testing, with further studies obtained as indicated.

### Echocardiographic assessment

All patients underwent transthoracic echocardiography (TTE) as part of the diagnostic stroke work-up. TEE was performed when TTE suggested valvular abnormalities or when a cardioembolic mechanism remained suspected. Echocardiographic examinations were performed and interpreted by European Association of Cardiovascular Imaging (EACVI)-accredited senior echocardiographers.

Nonbacterial thrombotic endocarditis (NBTE) was diagnosed if typical non-infectious valvular vegetations were present, characterized by at least one of the following^7^.

- sessile or mildly mobile echodense lesions likely composed of fibrin–platelet aggregates
- localization along valve closure lines, symmetric involvement of all three aortic cusps producing the characteristic “marantic kiss” sign^15^.
- uniform, non-destructive leaflet thickening without perforation, abscess, or cusp cavitation.

### Differential diagnosis

Exclusion of infective endocarditis (IE) was required prior to confirming NBTE. IE was considered unlikely when at least one of the following conditions was met:

- absence of bacterial and fungal infection, evidenced by serially negative blood cultures and lack of systemic infectious signs
- or clinical judgment favoring NBTE over infective endocarditis by a multidisciplinary endocarditis team.

### Follow-up protocol

All patients underwent TTE during routine stroke work-up. TEE was additionally performed in patients with suspected cardioembolic stroke mechanisms, morphologically abnormal valves or unexplained valvular dysfunction on TTE, or in younger patients undergoing evaluation for patent foramen ovale.

In patients diagnosed with NBTE, repeat echocardiography was generally performed approximately 7–10 days after initiation or modification of anticoagulation and subsequently according to clinical course until regression or disappearance of vegetations was documented. In selected patients, follow-up imaging was limited by palliative treatment goals, advanced oncologic disease, or death.

Follow-up assessments were scheduled for 12 months after the index event via telephone, documenting survival status, recurrent ischemic events, and functional outcomes.

For patients who could not be reached by telephone, vital status was additionally ascertained through the cancer registry of our institution.

Follow-ups were coordinated jointly between oncology, cardiology, and neurology teams.

### Clinical data collection

Demographic variables, cancer characteristics, cancer-directed therapies, vascular risk factors, and laboratory parameters were prospectively collected through systematic extraction from the hospital’s electronic medical record system (SAP-based hospital information system). No additional samples were collected specifically for the study. Laboratory analyses included markers routinely implicated in cancer-associated coagulopathy, specifically:

Complete blood count (hemoglobin, platelet count, leukocyte count), renal function (serum creatinine), coagulation parameters (INR, aPTT), inflammatory markers (C-reactive protein), and cardiac biomarkers (high-sensitivity troponin, NT-proBNP). D-dimer measurements were not systematically available in all patients because laboratory testing followed routine clinical care rather than a study-mandated biomarker protocol.

Exposure to anticoagulation was documented in detail, including indication, type of agent, dose, treatment modifications or switches, temporary interruptions, and adherence. Anticoagulation strategies, including any step-down from LMWH to DOACs, were individualized within a multidisciplinary framework and not guided by a predefined protocol.

Echocardiographic evaluations (TTE/TEE), when available, were reviewed to characterize vegetation morphology, valvular involvement, and hemodynamic consequences. Follow-up imaging was systematically assessed to categorize interval changes according to predefined criteria:

•Complete resolution – disappearance of all vegetations on follow-up imaging.

•Regression – measurable reduction in vegetation size or number without complete disappearance.

•Stable disease – no relevant change in size, appearance, or valvular involvement.

•Progression – increase in vegetation size/number, extension to additional valves, or new/worsening valvular dysfunction (regurgitation or stenosis).

### Stroke definition

Acute ischemic stroke was defined radiologically as either an acute diffusion-weighted imaging lesion with a corresponding apparent diffusion coefficient correlate on brain MRI or a new hypodensity on non-contrast CT corresponding to a new focal neurological deficit. If the initial diagnosis was based on non-contrast CT, timely follow-up MRI was performed for confirmation according to local protocols.

### Stroke etiology classification

Ischemic stroke etiology was classified per TOAST^16^. In addition, we adjudicated an MAH-related subtype for cases with infarcts involving all three vascular territories on neuroimaging in the absence of ≥ 50% culprit-artery stenosis, a major cardioembolic source other than NBTE, or another determined etiology.

### Statistical analysis

This was an exploratory prospective observational study. Continuous variables were evaluated for distribution and summarized as mean ± standard deviation or median with interquartile range, as appropriate. Group comparisons were performed using Welch’s t-test for normally distributed variables and the Mann–Whitney U test for non-normally distributed variables. Categorical variables were compared using Fisher’s exact test. No correction for multiple testing was applied due to the hypothesis-generating design. Statistical analyses were performed using IBM SPSS Statistics version 29.0 (IBM Corp., Armonk, NY, USA).

### Use of large language models

During the preparation of this work, the authors used ChatGPT (OpenAI) only for the linguistic editing of the final manuscript. The authors have reviewed and confirmed the validity of the text and take full responsibility for the content of the publication.

## Results

### Patient characteristics

**Fig. 1:**
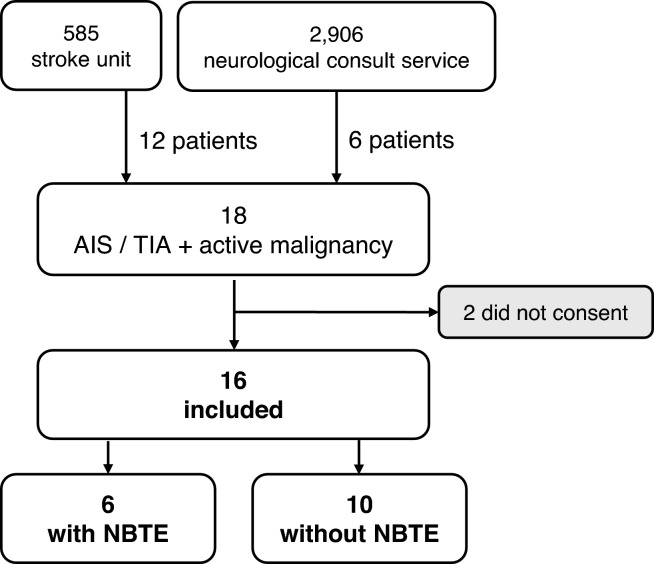
Patient flow chart for inclusion in the prospective study. Abbreviations: AIS: acute ischemic stroke; TIA: transient ischemic attack; NBTE: non-bacterial thrombotic endocarditis.

Patients were screened during treatment at our stroke unit and during evaluations by the neurology consultation service, including consultations on oncology wards. Eighteen patients with AIS/TIA and active malignancy were identified, of whom two declined study participation. No patients were excluded based on echocardiographic criteria. A total of 16 patients with active malignancy and AIS/TIA were included, of whom 6 (37.5%) were diagnosed with nonbacterial thrombotic endocarditis (NBTE) (Fig. 1). Baseline characteristics are shown in Table 1. Demographics and most routine laboratory values did not differ significantly between groups, except for hemoglobin, which was significantly lower in NBTE patients.

**Table 1:** Baseline characteristics of the study cohort.

Variable	All patients (n = 16)	NBTE (n = 6)	Non-NBTE (n = 10)	p-value
Demographics				
Age (years)	63.1 ± 11.0	57.5 ± 11.3	66.4 ± 9.8	0.12
Female sex, n (%)	62.5%	50.0%	70.0%	0.61
BMI (kg/m^2^)	24.0 ± 5.6	23.8 ± 5.6	24.2 ± 5.9	0.90
Height (cm)	167.4 ± 9.2	163.8 ± 11.1	169.5 ± 7.6	0.24
Weight (kg)	67.7 ± 18.0	64.3 ± 18.2	69.7 ± 18.5	0.58
Routine labs				
Creatinine (mg/dL)	1.0 ± 0.6	0.8 ± 0.4	1.1 ± 0.7	0.47
Hemoglobin (g/dL)	11.0 ± 3.1	8.8 ± 0.7	12.4 ± 3.3	0.007
Platelets (× 10^9^/L)	211.6 ± 125.8	185.5 ± 113.6	227.3 ± 136.0	0.54
White blood count (× 10^9^/L)	14.7 ± 15.9	19.0 ± 25.8	12.1 ± 5.8	0.42
INR	1.1 ± 0.2	1.2 ± 0.3	1.1 ± 0.2	0.53
CRP (mg/L), median (IQR)	50.3 (99.7)	42.4 (65.8)	71.8 (118.8)	0.87
Cardiac biomarkers				
hs-Troponin (ng/L), median (IQR)	30.0 (61.0)	73.5 (33.5)	21.0 (42.0)	0.33
NT-proBNP (pg/mL), median (IQR)	540.0 (4657.5)	2683.5 (5229.5)	515.5 (1352.2)	0.48
Echocardiography				
LVEF (%)	58.0 ± 5.5	56.0 ± 6.3	59.2 ± 4.9	0.27
LVEDD (mm)	44.6 ± 7.7	44.8 ± 6.3	44.4 ± 8.8	0.92
LVISd (mm)	10.4 ± 2.1	9.5 ± 2.2	10.9 ± 2.0	0.21
LVEDV (mL)	110.4 ± 23.6	112.5 ± 27.5	104.0 ± 2.8	0.69
LAVI (mL/m^2^)	32.5 ± 14.5	34.4 ± 20.8	31.5 ± 11.5	0.73
AV Vmax (m/s)	1.66 ± 0.8	2.12 ± 1.0	1.38 ± 0.42	0.14

### Cancer characteristics

Among the 16 patients with active malignancy and ischemic stroke, metastatic disease was present in 4/6 (66.7%) NBTE patients and 5/10 (50.0%) non-NBTE patients. Gastrointestinal/hepatobiliary malignancies were more common in the NBTE group (66.7% vs. 10.0%, OR 18.00, 95% CI 1.24–260.93; p = 0.036, Table 2). In the NBTE group, these cancers included cholangiocarcinoma, gallbladder carcinoma, and esophagogastric junction carcinoma, whereas pancreatic cancer was the only hepatobiliary malignancy observed in the non-NBTE group. No other individual tumor entity demonstrated a statistically significant association with NBTE.

**Table 2:** Clinical, cancer, and imaging characteristics in patients with active malignancy and stroke stratified by NBTE status.

Category	Variable	NBTE (n = 6)	Non-NBTE (n = 10)	OR	95% CI	p-value
Co-morbidities	Renal insufficiency	2/6 (33.3%)	6/10 (60.0%)	0.33	0.04–2.49	0.608
	Diabetes	0 (0.0%)	0 (0.0%)	–	–	–
	Arterial hypertension	4/6 (66.7%)	8/10 (80.0%)	0.50	0.06–4.24	0.604
	Liver disease/cirrhosis	0/0	1/10 (10%)	–	–	–
	COPD	1/6 (16.7%)	2/10 (20%)	0.8	0.06–11.3	1.000
On anticoagulation during index event		4/6 (66.7%)	1/10 (10%)	18.00	1.24–260.92	0.036
Cancer	Metastatic disease	4/6 (66.7%)	5/10 (50.0%)	2.00	0.24–16.36	0.63
	Respiratory malignancy	1/6 (16.7%)	3/10 (30.0%)	0.47	0.04–5.9	1
	GI/hepatobiliary cancer^†^	4/6 (66.7%)	1/10 (10.0%)	18.00	1.24–260.93	0.036
	Ovarian carcinoma^†^	2/6 (33.3%)	1/10 (10.0%)	4.00	0.77–82.69	0.518
	Hematologic malignancy	0/6 (0.0%)	2/10 (20.0%)	0.26	0.01–6.44	0.500
	Head and neck malignancy	0/6 (0.0%)	1/10 (10.0%)	0.41	0.02–10.29	1
Stroke presentation	Ischemic stroke	6/6 (100%)	9/10 (90.0%)	–	–	1.000
	TIA / amaurosis fugax	0/6 (0.0%)	1/10 (10.0%)	–	–	1.000
	Multi-territory embolic stroke	6/6 (100%)	6/10 (60.0%)	11.00	0.50–242.36	0.102
	Large-vessel occlusion	2/6 (33.3%)	0/10 (0.0%)	11.67	0.46–295.23	0.125
	Prior ischemic stroke	4/6 (66.7%)	1/10 (10.0%)	12.60	1.26–126.42	0.028
	Recurrent stroke after index	2/6 (33.3%)	2/10 (20.0%)	2.11	0.26–16.92	0.584
Thrombotic burden	Pulmonary embolism	1/6 (16.7%)	2/10 (20.0%)	0.8	0.06–11.32	1.000
	Deep vein thrombosis	3/6 (50.0%)	2/10 (20.0%)	4.00	0.43–37.11	0.299
Infective Endocarditis	IE (any vegetation)	0/6 (0.0%)	1/10 (10.0%)	0.49	0.02–13.92	1.000

^1^One patient had dual malignancy (acute myeloid leukemia with myelodysplasia-associated genetic changes and papillary thyroid carcinoma). Categorized as hematologic cancer based on highest thromboembolic risk; the thyroid carcinoma considered secondary.

^†^One patient with suspicion of either ovarian or colon carcinoma is counted in both gynecologic and GI/hepatobiliary categories GI/hepatobiliary cancers included cholangiocarcinoma, gallbladder carcinoma, and esophagogastric junction cancer in the NBTE group, and pancreatic cancer in the non-NBTE group.

### Stroke characteristics

**Fig. 2:**
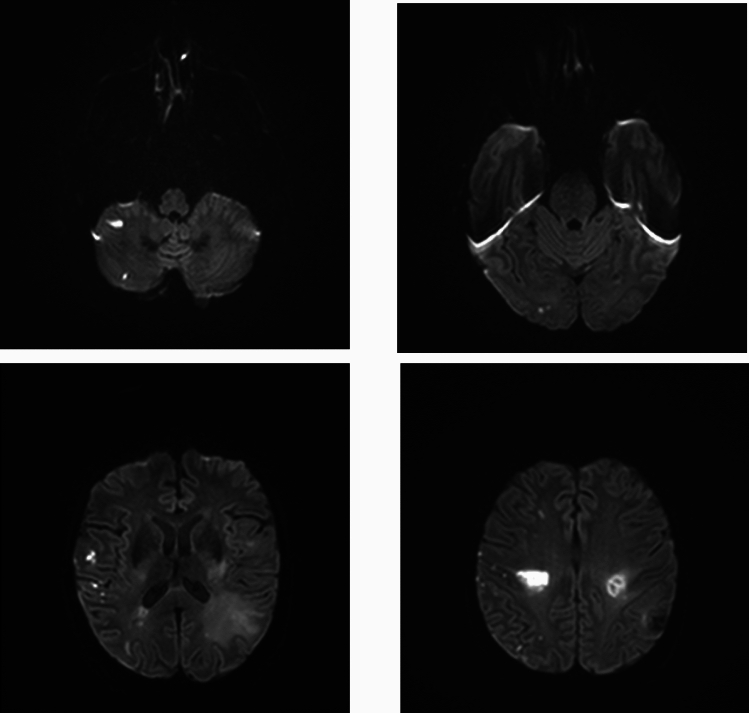
Axial diffusion-weighted MRI demonstrating multiple, metachronous ischemic infarctions in bilateral middle cerebral artery territories and in the posterior circulation (“three-territory sign”) in a patient with active malignancy and non-bacterial thrombotic endocarditis. Abbreviations: MRI, magnetic resonance imaging.

All patients experienced an ischemic cerebrovascular event. Multi-territory embolic infarction was observed in all NBTE patients (100%), compared with 60.0% of non-NBTE patients (OR 11.00, 95% CI 0.50–242.36; p = 0.102). Prior ischemic stroke was significantly more frequent in NBTE (66.7% vs. 10.0%; OR 12.60, 95% CI 1.26–126.42; p = 0.028). Recurrent stroke occurred more often in NBTE as well, although this difference was not statistically significant (50% vs. 20%; OR 4.00, 95% CI 0.43–37.11; p = 0.299). Neurological severity at admission and discharge (NIHSS) and functional outcomes (mRS) did not differ significantly between groups (Fig. 2).

### Anticoagulation exposure and breakthrough AIS

At the time of the index stroke, 4/6 (66.7%) NBTE patients were receiving direct oral anticoagulants (DOACs), while none were treated with low-molecular-weight heparin (LMWH). Indication for anticoagulation was a history of deep vein thrombosis in three of these patients; in the fourth, the indication for ongoing anticoagulation was unclear. Breakthrough AIS occurred under DOAC therapy, leading to a subsequent switch to LMWH in several cases (Table 3). Across all NBTE cases, valvular vegetation regression was observed in 3/ 4 (75%) cases under LMWH. The fourth patient showed no clear regression in follow-up TEEs under LMWH. No breakthrough AIS occurred under LMWH.

**Table 3:** Anticoagulation and follow-up outcomes of NBTE patients.

Patient	Baseline stroke presentation	Baseline echocardiography (TTE/TEE)	Anticoagulation at baseline	Follow-up echocardiography course (TTE/TEE)	Outcome
P1	AIS with bilateral MCA, PCA, and cerebellar infarcts (multi-territory). Symptoms: worsening right hemiparesis and aphasia. NIHSS 7 → 7, mRS 4 → 4. CT + , CTA + , MRI + . History of three recurrent embolic events. Mechanism: cardioembolic	TEE required for confirmation of typical NBTE echo findings (multiple aortic valve vegetations consistent with NBTE and severe aortic regurgitation)	Apixaban for prior DVT → switched to argatroban and eventually to unfractionated heparin	Month 1: Complete regression of vegetations; switched back to apixaban. Month 2: Left M1 occlusion treated with mechanical thrombectomy; switched to LMWH. Month 3: Transient aphasia → LMWH dose increased. Months 4–5: DOAC restarted due to pain at injection site→ recurrent M1 stenosis and NBTE recurrence with new mitral involvement 10 days later; moderate MR and moderate AS	Death
P2	AIS with bilateral ACA/MCA/PCA involvement, no focal deficit. NIHSS 2 → 2, mRS 2. CT + , MRI + . Prior PCA stroke with normal CTA/Doppler. Mechanism: cardioembolic	TTE sufficient for typical NBTE echo findings (Aortic valve NBTE with noninfectious vegetations)	No anticoagulation → LMWH initiated	Months 4–5: Complete resolution of vegetations. Month 6: Switched to rivaroxaban. Month 7: NBTE recurrence on rivaroxaban with new ventricular-side vegetations and moderate aortic regurgitation	Death due to progressive malignant disease
P3	AIS with extensive multi-territory infarcts (bilateral MCA; right PCA + ACA; bilateral cerebellar). Symptoms: left motor aphasia syndrome, mild hemiparesis, dysarthria. NIHSS 4 → 4, mRS ~ 1 → 1. CT + , CTA + , MRI + . Cardioembolic mechanism. Silent prior infarcts	TEE required for confirmation of typical NBTE echo findings (NBTE involving mitral and aortic valves)	Apixaban for recent DVT → switched to LMWH	Week 2: Mitral vegetation regressed; aortic lesion unchanged	Death due to recurrent embolic stroke
P4	AIS with left ACA/MCA and right PCA involvement. Symptoms: consciousness disturbance, aphasia, right hemineglect, motor aphasia syndrome. NIHSS 7 → 4, mRS 3 → 3. CT + , CTA + , MRI + . Large-vessel stenoses present; mechanism considered embolic/paraneoplastic/ESUS	TTE sufficient for typical NBTE echo findings (Small aortic vegetations visible on TTE)	No anticoagulation → apixaban at discharge	Month 2: Progressive aortic vegetations without significant AR/AS. Month 3: No residual vegetation (regression vs silent embolization)	Death due to progressive malignant disease
P5	AIS with bilateral MCA and PCA infarcts and right cerebellar involvement. Symptom: left quadrantanopsia. NIHSS 1 → 1, mRS 1 → 1. MRI + . Mechanism: cardioembolic. History of DVT and arterial thrombosis (popliteal artery)	TEE required for confirmation of typical NBTE ehco findings (Tricuspid aortic valve with broad-based vegetations on all cusps; moderate–severe AR)	Rivaroxaban → switched to LMWH	Month 1–2: Stable vegetations with progression to severe AR, LV dilation, and retrograde diastolic aortic flow. Month 3: Slight regression of vegetations, persistent severe AR	Alive
P6	AIS with bilateral MCA infarcts. Symptoms: worsening pre-existing leg paresis, left motor aphasia syndrome, dysarthria. NIHSS 5, mRS ~ 4. CT + , MRI + , Doppler + . Mechanism: cardioembolic. Prior recurrent infarcts	TTE sufficient for typical NBTE echo findings (Aortic and mitral NBTE vegetations)	Apixaban → bridged to LMWH for biopsy → switched to edoxaban	Persistent aortic and mitral vegetations on serial TEE. Recurrent embolic strokes despite anticoagulation	Death due to recurrent embolic stroke

Abbreviations: AIS, acute ischemic stroke; MCA, middle cerebral artery; ACA, anterior cerebral artery; PCA, posterior cerebral artery; CT, computed tomography; CTA, CT angiography; MRI, magnetic resonance imaging; NIHSS, National Institutes of Health Stroke Scale; mRS, modified Rankin Scale; NBTE, nonbacterial thrombotic endocarditis; AR, aortic regurgitation; AS, aortic stenosis; MR, mitral regurgitation; LV, left ventricle/left ventricular; DVT, deep vein thrombosis; LMWH, low-molecular-weight heparin; DOAC, direct oral anticoagulant; ESUS, embolic stroke of undetermined source; MAS, motor aphasia syndrome; TEE, transesophageal echocardiography; TTE, transthoracic echocardiography.

In contrast, under DOAC therapy persistent or progressive NBTE—including all cases of recurrent embolism—occurred in 5/6 (83%) patients. The only apparent resolution of vegetations, observed while a patient remained on DOAC, occurred after a period during which the vegetations had previously progressed. Because no change in anticoagulant therapy was made and no intervening clinical embolic event was recorded, silent embolization could not be excluded as an alternative explanation for the later absence of detectable vegetations.

No unequivocal regression of NBTE was observed under DOAC therapy.

No major bleeding events occurred during follow-up in either anticoagulation group. Minor bleeding events were not systematically collected as they did not lead to hospitalization, treatment discontinuation, or transfusion.

### Echocardiographic findings

TEE was performed in 11 of 16 patients (68.8%), while 5 patients underwent transthoracic echocardiography (TTE) only. NBTE predominantly involved the aortic valve (100%), with concomitant mitral involvement in 50%.

Serial echocardiography suggested differing patterns of vegetation evolution under different anticoagulation regimens:

- Regression of valvular vegetations occurred in 75% of the cases under LMWH, while
- In this small observational cohort, progression (including new mitral involvement and worsening aortic regurgitation), was observed only in patients receiving DOAC therapy, including cases of re-exposure to DOACs after prior regression on LMWH. However, treatment allocation was non-randomized and findings must be interpreted cautiously

**Fig. 3:**
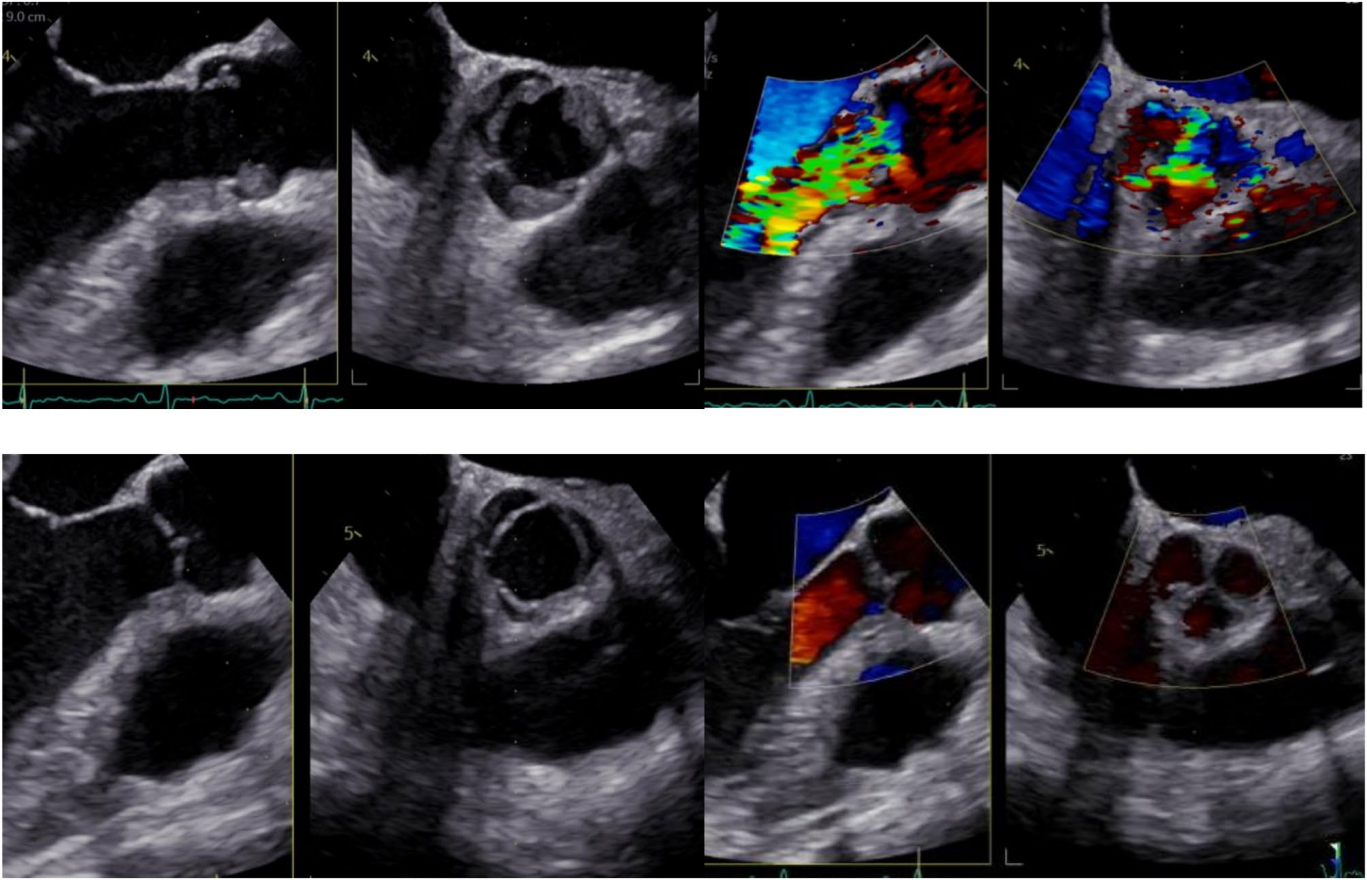
TEE changes in relation to anticoagulation in one NBTE patient. TEE revealing NBTE affecting the aortic valve, accompanied by severe aortic regurgitation in a patient receiving DOAC. Anticoagulation was then transitioned to LMWH, after which serial TEE examinations demonstrated complete regression of the aortic vegetations, including resolution of the regurgitation. Abbreviations: NBTE, nonbacterial thrombotic endocarditis; TEE, transesophageal echocardiography; LMWH, low-molecular-weight heparin.

Evaluation of the echocardiographic data by a single experienced echocardiographer revealed a 50% (3 of 6 cases) accuracy of TTE alone in raising a high suspicion of NBTE, whereas in half of the cases a TEE was required to establish the diagnosis (Fig. 3).

### Oncologic treatment and disease course in patients with NBTE

Patients with NBTE received heterogeneous systemic oncologic therapies depending on tumor entity and stage, including platinum-based chemotherapy, immune checkpoint inhibitors, targeted therapies, and hypomethylating agents. Most patients had advanced or metastatic malignancy and received multiple sequential lines of therapy, reflecting treatment-refractory disease.

A structured overview of oncologic treatments and clinical disease course in patients with NBTE is provided in Table 4. Overall, most patients demonstrated progressive or advanced disease during follow-up. Persistent tumor progression was common in patients with ongoing or recurrent thromboembolic events. However, due to the observational design and frequent changes in anticoagulation therapy, no causal relationship between oncologic response and NBTE course can be established.

**Table 4:** Oncologic treatment, disease course, and NBTE-related outcomes in patients with NBTE.

Patient	Cancer entity	Oncologic treatment	Disease course	Embolic/vascular course
P1	Metastatic ovarian carcinoma (FIGO IIIA)	Carboplatin/paclitaxel/bevacizumab → carboplatin + PLD → bevacizumab maintenance → olaparib	Advanced metastatic, long-term treated disease; persistent malignant activity	Highly dynamic NBTE with regression under heparin, recurrence under DOAC; recurrent embolic strokes; death
P2	Metastatic AEG tumor (cerebral/meningeal/lymphatic)	Nivolumab + 5-FU/folinic acid	Rapidly progressive metastatic disease	NBTE regression under LMWH, recurrence under DOAC; embolic complications; death
P3	Metastatic NSCLC with brain metastases	Multi-line therapy: Osimertinib → chemo-immunotherapy → pemetrexed → rechallenge osimertinib → gemcitabine/carboplatin → amivantamab-based therapy	Highly treatment-refractory progressive disease	Persistent NBTE with incomplete regression; recurrent embolic strokes; death
P4	Cholangiocarcinoma	Capecitabine → cisplatin/gemcitabine + durvalumab	Advanced biliary malignancy; progressive clinical course	NBTE not present initially; new vegetation during course with later disappearance; embolic event pattern vs. regression; death
P5	Suspected ovarian vs colon carcinoma with peritoneal carcinomatosis	Carboplatin + paclitaxel weekly → dose reduction due to complications	Advanced metastatic disease with complications (ascites, bleeding, embolization procedures)	Persistent NBTE with progressive valvular destruction and severe AR; alive
P6	AML (MPN-related) + papillary thyroid carcinoma	Azacitidine → venetoclax → decitabine	Hematologic malignancy with ongoing active disease	Persistent NBTE without regression; recurrent embolic stroke; death

Abbreviations: 5-FU, 5-fluorouracil; AEG, adenocarcinoma of the esophagogastric junction; AML, acute myeloid leukemia; AR, aortic regurgitation; FIGO, International Federation of Gynecology and Obstetrics; MPN, myeloproliferative neoplasm; NBTE, non-bacterial thrombotic endocarditis; NSCLC, non-small cell lung cancer; PLD, pegylated liposomal doxorubicin.

### Outcomes

Twelve-month vital status was available for all patients (n = 16). Mortality was 31.25% (5/16) at 1 month, 62.5% (10/16) at 6 months, and 81.25% (13/16) at 12 months. Three patients (18.75%) were alive at 12 months: two were living at home (one with minor neurological deficits and one requiring assistance with activities of daily living but able to walk), and one was receiving hospice care.

Probable MAH was present in all patients who died within 1 month (5/5, 100%) and in 7/10 (70%) of those who died within 6 months. NBTE was observed in 2/5 (40%) patients who died within 1 month and 5/10 (50%) who died within 6 months. Of the three patients alive at 12 months, 1/3 (33%) had probable MAH with NBTE and was receiving hospice care. No patient developed clinical evidence of infective endocarditis during follow-up.

## Discussion

NBTE, a severe manifestation of MAH, is an important but probably under-recognized cause of AIS in patients with cancer. However, epidemiological data on NBTE prevalence in this context is scarce and highly variable. We conducted, to our knowledge for the first time, a single-center prospective study including patients with AIS or TIA and active malignancy, using a standardized screening protocol for NBTE. Our main findings are:

1. NBTE was identified in 37.5% of this selected prospective cohort undergoing systematic echocardiographic evaluation. Among the NBTE cases, 4 of 6 patients (66.7%) had gastrointestinal or hepatobiliary malignancies, highlighting a predominance of these cancer types in association with NBTE at our institution.
2. All NBTE cases showed a radiological pattern with multi-territory infarcts.
3. A quarter of the patients with a stroke pattern suggestive of MAH/NBTE presented with subtle and/or non-focal neurological symptoms that are easy to overlook in routine care.
4. The data from our cohort suggests lower rates of clinical and radiological progression in patients treated with LMWH than DOACs. However, interpretation is limited by small patient numbers, treatment-selection bias, and the observational design of the study.
5. Prognosis was poor: 10 out of 11 patients (90.9%) with AIS due to MAH/NBTE died within 12 months.

### Prevalence of NBTE

Epidemiological data on NBTE remain highly variable across studies. González-Quintela et al. identified ten cases of NBTE among 1,640 adult autopsies, corresponding to a prevalence of 1.25% in patients with malignancy compared with 0.2% in those without cancer^17^. A second autopsy-based analysis including 3,426 patients with cancer and 500 individuals with cerebrovascular disease reported NBTE as the etiology of stroke in 18% of cases, underscoring its potential relevance in cancer-associated cerebrovascular events^18^.

In one prospective echocardiographic study of 200 patients with active cancer, cardiac valvular vegetations consistent with NBTE were detected in 19%^9^. Data on the prevalence of NBTE in patients with cancer-associated AIS are scarce and derived exclusively from retrospective studies. In a study of 51 cancer patients with cerebral ischemia, 18% were diagnosed with cancer-associated NBTE, a proportion consistent with that observed in our cohort^11^. In another retrospective cohort of 245 patients with stroke and cancer, only 20 patients (8.2%) were identified as having NBTE; however, TEE—the diagnostic gold standard—was performed in only 60.4%, likely leading to an underestimation of true prevalence^10^.

Taken together, available evidence indicates that NBTE may be substantially underdiagnosed, particularly in patients with active malignancy and AIS, and that its true prevalence might be higher than reported in routine clinical practice. In our cohort, NBTE was identified in 37.5% of patients, suggesting that it is a frequent mechanism of cancer-associated stroke. At our stroke unit, AIS related to active malignancy accounted for approximately 2.6% of cases, and NBTE was identified in roughly 0.7% of all ischemic stroke cases. Inclusion of in-hospital strokes from other departments—particularly oncology—would likely increase this proportion; in line with the existing literature and based on the available data, we estimate the center-wide frequency of AIS related to active malignancy to be ~ 4% and the frequency of AIS due to NBTE to be ~ 1,4%, although we lack complete coverage of all departments.

### Radiologic ischemic stroke patterns

All patients diagnosed with NBTE in our cohort had multiple acute infarcts involving all three major vascular territories (carotid artery territories bilaterally plus vertebro-basilar artery territories, “three-territory sign”). However, this pattern is not specific for NBTE but rather a frequent feature of MAH-related stroke in general. In our study, 11 out of 16 patients (69%) presented with a three-territory pattern and 6 out of these 11 patients (55%) were ultimately diagnosed with NBTE. Of note, while a positive “three-territory-sign” has frequently been linked to MAH and NBTE^19^, a retrospective analysis of 84 MRI scans from patients with AIS and cancer-related NBTE found multi-territory infarction in most, but not all cases^20^. Taken together, these findings suggest that the three-territory pattern should prompt a systematic search for an embolic source, including infectious endocarditis and NBTE, as well as for an active malignancy if no convincing cardiac or arterial source is identified. At the same time, the absence of this pattern and the presence of only single or limited lesions do not exclude NBTE or MAH as the underlying cause of stroke.

### Non-focal clinical manifestation

3 of the 11 patients (27%) who were later found to have multiple infarctions in all three vascular territories did not present with focal neurological deficits but instead with altered mental status or no neurological symptoms at all. All of these patients were treated on oncology wards for their underlying malignancy. In these cases, the diagnosis of MAH/NBTE was only suspected after brain MRI revealed multiple acute infarcts. Such presentations are likely to be overlooked in routine care, where fluctuating cognition or nonspecific symptoms are often attributed to metabolic or treatment-related causes. Cerebral MRI and neurological assessment should therefore be considered generously in patients with active malignancies, even when focal deficits are absent or subtle.

### Treatment

Only limited retrospective data exist on the management of NBTE. Although heparin is recommended over no anticoagulation, this is based solely on a 2C recommendation^21^. Previous studies support the effectiveness of unfractionated heparin in preventing recurrent thromboembolism in paraneoplastic syndromes^6,22–26^. Although LMWH generally appears effective, unresponsiveness has been reported in patients with MAH, in whom unfractionated heparin has ultimately proven successful^6^. Vitamin K antagonists are not recommended in NBTE, due to the possible presence of procoagulant non-vitamin K–dependent mechanisms^6,22,24,25^. Although some studies have shown the noninferiority of edoxaban, apixaban, and rivaroxaban to dalteparin regarding recurrence of VTE in cancer patients^27–29^, existing data on DOAC effectiveness in NBTE shows risk of recurrence of thromboembolic events. Moreover, edoxaban and rivaroxaban have been associated with a significantly higher risk of major bleeding compared with dalteparin in patients with active malignancy^6,13,14,30^. In line with existing literature, we observed fewer recurrent embolic events in patients treated with heparin-based therapy compared with DOACs, though these findings must be interpreted with caution given the small number of NBTE patients in our study (n = 6). Notably, there was one case of stroke due to NBTE despite anticoagulation with a DOAC, with complete resolution after switching to LMWH and documented recurrence after re-switching to a DOAC. A second case experienced complete resolution of the vegetation on LMWH, with recurrence after switching to a DOAC. A similar case has previously been reported by our group, in which NBTE lesions present under apixaban completely resolved after 5 weeks of LMWH therapy, recurred when apixaban was restarted, and again resolved following another 4 weeks of LMWH, illustrating the dynamic response of NBTE to different anticoagulation strategies^14^. Decisions regarding the choice of agent and duration of anticoagulation should consider vegetation evolution on follow-up imaging, comorbidities, prognosis, bleeding risk, and patients’ preferences ideally within a multidisciplinary care framework. Larger prospective studies are needed to define optimal anticoagulation strategies in malignancy associated NBTE.

### Prognosis

Prognosis in patients with cancer and stroke is generally poor^31–35^. In addition, existing literature has linked multi-territory infarctions suggestive of MAH/NBTE with reduced survival^33^. In line with previous reports of limited mean survival time, 90.9% of patients with AIS and MAH and/or NBTE died within 1 year. Almost all patients with NBTE showed progression before death, indicating that thromboembolic events may have contributed to the high mortality. However, interpretation is limited by the absence of a control group with comparable cancer stages but without AIS. It thus remains unclear to what extent mortality was driven by advanced malignancy—with MAH as a potential marker of progressive disease—versus the cerebrovascular event and its complications. Nevertheless, these findings should not lead to therapeutic nihilism. With ongoing advances in cancer therapy and growing experience in the management of MAH/NBTE, outcomes in selected patients may improve. Moreover, many patients in our cohort initially had only mild-to-moderate neurological deficits, and some experienced several months of good quality of life at home after the index event.

### Limitations

This study has several limitations. First, it is a single-center analysis with a relatively small sample size, which limits the generalizability of our findings and increases the risk that the results are driven by local practice patterns and the focus on specific cancer entities. The tertiary-care setting and specialized cardio-oncology expertise at our institution may additionally limit external validity. Second, there is a potential inclusion bias, as patients with an unclear stroke etiology might have been more likely to be enrolled than those with a clearly identifiable cause unrelated to NBTE or MAH, although we aimed to include all patients with active malignancy and AIS/TIA. This may partly explain the high prevalence of NBTE in our cohort. The small sample size substantially limits statistical power and increases the possibility that observed associations were influenced by chance. Accordingly, all findings should be interpreted as exploratory and hypothesis-generating rather than definitive. However, to our knowledge, this is the first prospective study on NBTE in patients with malignancy-related AIS, and the use of a systematic diagnostic work-up with dedicated screening for NBTE provides robust, hypothesis-generating data for future research.

### Open questions

Further prospective studies are needed to establish the prevalence of NBTE in larger cohorts of patients with AIS and active malignancy, and to address several important open questions regarding diagnostic and therapeutic strategies:

- Are there clinically relevant, clearly defined subgroups of patients, such as those in whom cancer is a bystander versus those with MAH–related stroke, and do these subgroups differ in prognosis and response to treatment?
- Which clinical, laboratory, and imaging markers reliably identify MAH and NBTE (e.g., markedly elevated D-dimer levels, a “three-territory sign” infarction pattern, typical echocardiographic patterns such as the “marantic kiss” sign), and how can these markers be incorporated into diagnostic algorithms and risk stratification?
- In patients with AIS and suspected MAH but no initially detectable NBTE, does systematic and/or serial echocardiography improve NBTE detection, and is systematic screening for VTE warranted in this population?
- Which anticoagulant agent and what duration are most effective and safe for secondary prevention?
- What is the prevalence of NBTE across different cancer types, and is broad echocardiographic screening feasible to enable early detection and treatment before stroke occurs?

## Conclusion

In patients with malignancy-related AIS or TIA, prevalence of NBTE is high and constitutes an important embolic source. Early detection through stroke pattern recognition and targeted echocardiography may provide a critical therapeutic window to reduce recurrent cardioembolic events. Future multi-center collaborations are warranted to confirm these findings and refine diagnostic and therapeutic strategies in this high-risk population.

## Data Availability

The data that support the findings of this study are available from the corresponding author upon reasonable request. Due to ethical and privacy restrictions related to patient data, the datasets are not publicly available.

## Abbreviations

5-FU: 5-Fluorouracil
ACA: Anterior cerebral artery
AEG: Adenocarcinoma of the esophagogastric junction
AIS: Acute ischemic stroke
AML: Acute myeloid leukemia
AR: Aortic regurgitation
AS: Aortic stenosis
CT: Computed tomography
CTA: Computed tomography angiography
DOAC: Direct oral anticoagulant
DVT: Deep vein thrombosis
DZHK: Deutsches Zentrum für Herz-Kreislauf-Forschung (German Center for Cardiovascular Research)
FEACVI: Fellow of the European association of cardiovascular imaging
FESC: Fellow of the European society of cardiology
FIGO: International federation of gynecology and obstetrics
LMWH: Low-molecular-weight heparin
M1: First segment of the middle cerebral artery
MCA: Middle cerebral artery
MPN: Myeloproliferative neoplasm
MR: Mitral regurgitation
MRI: Magnetic resonance imaging
mRS: Modified rankin scale
NBTE: Non-bacterial thrombotic endocarditis
NIHSS: National Institutes of Health Stroke Scale
NSCLC: Non-small cell lung cancer
PCA: Posterior cerebral artery
PLD: Pegylated liposomal doxorubicin
TEE: Transesophageal echocardiography
TTE: Transthoracic echocardiography
